# New insights into the treatment of nasopharyngeal carcinoma in children, adolescents, and young adults: a retrospective study

**DOI:** 10.3389/fimmu.2026.1765851

**Published:** 2026-02-27

**Authors:** Mengwei Ren, Jing Tian, Mengyuan Han, Shiran Sun, Kai Wang, Runye Wu, Meng Yuan, Junlin Yi, Fei Ma, Sidan Li

**Affiliations:** 1 Department of Medical Oncology, National Cancer Center/National Clinical Research Center for Cancer/Cancer Hospital, Chinese Academy of Medical Sciences and Peking Union Medical College, Beijing, China; 2 Hematology Center, Beijing Key Laboratory of Pediatric Hematology Oncology; National Key Discipline of Pediatrics (Capital Medical University); Key Laboratory of Major Diseases in Children, Ministry of Education; Beijing Children’s Hospital, Capital Medical University, National Center for Children’s Health, Beijing, China; 3 Department of Pediatrics, Beijing Shijitan Hospital, Capital Medical University, Beijing, China; 4 Department of Radiation Oncology, National Cancer Center/National Clinical Research Center for Cancer/Cancer Hospital, Chinese Academy of Medical Sciences and Peking Union Medical College, Beijing, China

**Keywords:** adolescent and young adult, children, immune checkpoint inhibitors, induction chemotherapy, nasopharyngeal carcinoma, retrospective study

## Abstract

**Objective:**

To evaluate the efficacy, safety, and survival impacts of diverse induction chemotherapy regimens (including combination therapy with immune checkpoint inhibitors [ICIs]), radiotherapy modalities, and consolidation therapy in children, adolescents, and young adults (CAYA) with locally advanced or metastatic nasopharyngeal carcinoma (NPC).

**Methods:**

This multicenter retrospective study analyzed 102 CAYA NPC patients (aged 6–24 years; stage III–IVB) from two Chinese centers (January 2011–October 2024). All received induction therapy followed by concurrent chemoradiotherapy (CCRT), radiotherapy combined with concurrent anti-EGFR therapy, or radiotherapy alone, with select cases receiving consolidation (median follow-up: 22 months).

**Results:**

GP and TPF induction achieved higher objective response rates (ORR) vs. TP (GP: 68.0% vs. TP: 31.6%, P = 0.005; TPF: 89.5% vs. TP: 68.0%, P < 0.001), though no significant difference in long-term survival was observed. ICIs + chemotherapy (n=15) improved ORR (93.3% vs. 53.3%, P = 0.013) though without a demonstrable difference in survival metrics at this follow-up. In patients achieving partial response (PR) post-induction, CCRT/anti-EGFR therapy + radiotherapy improved 1-year progression-free survival (PFS: 94.5% vs. 50.0%, P < 0.001) and distant metastasis-free survival (DMFS: 97.4% vs. 50.0%, P < 0.001). For stable disease (SD) patients, multimodal therapy increased 5-year overall survival (OS: 100% vs. 66.7%, P = 0.046). Consolidation therapy (n=24) in locally advanced NPC was associated with clinical stage (P = 0.001) but not survival (P > 0.05).

**Conclusion:**

TPF/GP regimens improved short-term responses with manageable toxicity. The addition of ICIs enhanced objective response rates, though survival benefits were not assessable within the limited follow-up period. CCRT demonstrated survival advantages over radiotherapy alone, especially in PR patients, while consolidation therapy showed limited benefit except in advanced subgroups. These findings, generated from a retrospective analysis, highlight the need for personalized strategies and warrant validation in larger prospective trials.

## 1 Introduction

Nasopharyngeal carcinoma (NPC) is a malignancy of epidermoid origin distinguished from other head and neck cancers by its unique histological, epidemiological, and biological characteristics (1). Studies have demonstrated that Epstein-Barr virus (EBV) infection serves as a significant causative factor in the pathogenesis of NPC (2). Although NPC represents a relatively low proportion (1-3%) of malignancies in children, adolescents, and young adults (CAYA) (3), its clinical presentation and diagnostic challenges in this population warrant particular attention. CAYA NPC patients frequently present with non-specific symptoms such as rhinorrhea and nasal obstruction. The anatomically concealed location of NPC lesions, combined with the physiological growth phase of adolescence, often leads to misinterpretation of pathological manifestations as normal growth-related symptoms. Consequently, approximately 80% of CAYA NPC cases are diagnosed at locally advanced stages, with significantly more advanced disease compared to adult patients at initial presentation (4). This clinical pattern not only correlates strongly with diagnostic delays, but also directly impacts treatment strategy formulation and long-term prognosis of patients.

The current standard treatment for advanced CAYA NPC patients involves platinum-based induction chemotherapy combined with concurrent chemoradiotherapy (CCRT), which achieves favorable prognoses in most patients (5). However, the optimal therapeutic strategy for this population remains controversial. The triple-drug regimen (TPF)—adding docetaxel to the traditional cisplatin and 5-fluorouracil (PF) regimen—has demonstrated superior efficacy in adults with locally advanced NPC (6), with its application gradually extending to CAYA populations. Concurrently, studies from Sun Yat-sen University Cancer Center revealed that the gemcitabine plus cisplatin (GP) regimen improved 3-year recurrence-free survival (RFS) and overall survival (OS) rates in adults with locally advanced NPC to 80.2% and 90.3%, respectively (7). Nevertheless, approximately 30% of advanced patients still experience recurrence or metastasis after primary treatment, accompanied by severe toxicities (e.g., xerostomia, dysphagia) (8, 9).

Breakthroughs in immunotherapy have opened new avenues for NPC treatment. The high expression of PD-L1 in NPC tissues mediates tumor immune evasion via the PD-1/PD-L1 signaling pathway, providing a mechanistic rationale for immune checkpoint inhibitors (ICIs) application (10). Clinical trials report that nivolumab monotherapy achieves a 1-year OS rate of 59% in recurrent/metastatic NPC patients, with only 22% incidence of severe adverse events(AEs) (11). Notably, a phase III trial conducted by Sun Yat-sen University demonstrated that combining PD-1 inhibitors with chemoradiotherapy enhances 3-year disease-free survival by 10% and reduces recurrence/metastasis risk by 40% in locally advanced patients (12).

However, these findings primarily derive from adult studies, while robust evidence specific to the CAYA population—particularly regarding ICIs combination therapies—remain scarce. To date, limited research has explored the impact of standard chemotherapy combined with ICIs on radiotherapy dosing and long-term survival in CAYA patients (13), and safety profiles of ICIs in this population have not been fully characterized. This retrospective study systematically reviews treatment responses and prognoses in advanced CAYA NPC patients, aiming to optimize pharmacological strategies and elucidate the efficacy-safety profile of ICIs-based combination therapies, thereby contributing evidence-based insights for precision medicine in this unique cohort.

## 2 Literature and methods

### 2.1 General information

A retrospective analysis was conducted on 102 CAYA NPC patients treated at the Cancer Hospital of the Chinese Academy of Medical Sciences and Beijing Children’s Hospital between January 2011 and October 2024. Inclusion criteria were defined as: (1) age 6–24 years; (2) histopathologically confirmed nasopharyngeal carcinoma; (3) locally advanced or advanced disease classified as stage III to IVB according to the American Joint Committee on Cancer (AJCC) 8th edition staging system (2017). Exclusion criteria included absence of induction therapy prior to radiotherapy.

### 2.2 Treatment

Following confirmed pathological diagnosis, clinical staging, and exclusion of therapeutic contraindications, all patients initially underwent induction therapy. Subsequent treatment modalities included CCRT, radiotherapy combined with concurrent anti-EGFR therapy, or radiotherapy alone, with a subset of patients receiving consolidation therapy. The induction therapy primarily consisted of platinum-based doublet or triplet regimens, with 15 patients also received concurrent PD-1 inhibitor treatment. The specific induction chemotherapy regimens were as follows: (1) GP regimen: Gemcitabine 1.0g/m² intravenously on days 1 and 8, plus Cisplatin 75-80mg/m² intravenously on day 1, every 3 weeks (with Nedaplatin or Lobaplatin substituted in cases of Cisplatin contraindication). (2) TPF regimen: Docetaxel 60mg/m² or Albumin-bound Paclitaxel 260mg/m² intravenously on day 1, plus Cisplatin 60mg/m² intravenously on day 1, plus Fluorouracil 600mg/(m²·d) via micro-infusion pump on days 1-5, every 3 weeks. (3) TP regimen: Docetaxel 60mg/m² or Albumin-bound Paclitaxel 260mg/m² intravenously on day 1, plus Cisplatin 75-80mg/m² intravenously on day 1, every 3 weeks. (4) PF regimen: Cisplatin 75-80mg/m² intravenously on day 1, plus Fluorouracil 750mg/(m²·d) via micro-infusion pump on days 1-5, every 3 weeks.

This study employed multiple ICIs administered at evidence-based dosages per manufacturer prescribing information or standardized clinical trial protocols. Pembrolizumab was dosed at 2 mg/kg (maximum 200 mg) intravenously every 3 weeks, while camrelizumab was administered at 3 mg/kg (maximum 240 mg) intravenously every 3 weeks. All PD-1 monoclonal antibody regimens maintained dose intensities below established maximum adult thresholds.

All patients commenced CCRT or radiotherapy combined with concurrent anti-EGFR therapy following varying degrees of induction therapy. The delineation of radiotherapy target volumes was as follows: Gross Tumor Volume (GTV) included the nasopharyngeal primary tumor as indicated by imaging, nasoendoscopy, and clinical examination; GTV rpn for retropharyngeal metastatic lymph nodes; GTV nd for cervical metastatic lymph nodes; Clinical Target Volume (CTV) 1 extended 0.5~1 cm beyond GTV and GTV rpn, encompassing surrounding high-risk structures; CTV2 extended 0.5~1 cm beyond CTV1, including surrounding low-risk structures; Planning Target Volume (PTV) extended 2~5 mm beyond the corresponding target volumes. The radiotherapy dose was uniformly prescribed as 70 Gy in 33 fractions to Planning Gross Tumor Volume (PGTV), PGTV rpn, and PGTV nd (with efficacy evaluated at 40 Gy and dose reduced to 60 Gy for complete responders); 60 Gy in 33 fractions to PTV1; and 50 Gy in28 fractions to PTV2. During radiotherapy, some patients received varying courses of platinum-based concurrent chemotherapy, while others received concurrent anti-EGFR therapy with Nimotuzumab. The specific concurrent chemotherapy/anti-EGFR therapy regimens were as follows: (1) Cisplatin 80 mg/m² intravenously on day 1, every 3 weeks; (2) Lobaplatin 50 mg/m² intravenously on day 1, every 3 weeks; (3) Nedaplatin 80 mg/m² intravenously on day 1, every 3 weeks; (4) Nimotuzumab 200mg intravenously on day 1, weekly.

Twenty-four patients received subsequent consolidation therapy, with 5 patients receiving PD-1 inhibitor ± Nimotuzumab/Capecitabine (650mg/m², twice daily, for 1 year), and the remaining 19 patients receiving GP, TPF, TP, PF, or Capecitabine regimens, with specific dosing as previously described. All patients entered the follow-up phase upon completion of radiotherapy or consolidation therapy and were monitored until disease recurrence or distant metastasis occurred. **Table 1** provides a schematic overview of the multimodal treatment approaches used in this study.

**Table 1:** Overview of treatment pathways for CAYA NPC patients.

Treatment phase	Regimen/modality	Details	Patients (n)
Induction Therapy	Chemotherapy alone	TPF, GP, TP, or PF regimens (as detailed in text)	87
	Chemotherapy + ICIs	TP/GP + PD-1 inhibitor (± nimotuzumab)	15
Primary Radiotherapy	Concurrent Chemoradiotherapy (CCRT)	Radiotherapy + concurrent platinum-based chemotherapy	61
	Radiotherapy + Anti-EGFR	Radiotherapy + concurrent nimotuzumab	24
	Radiotherapy Alone	Definitive radiotherapy only	15
Consolidation Therapy (Post-Radiotherapy)	Chemotherapy	GP, TPF, TP, PF, or capecitabine	19
	ICIs-based Therapy	PD-1 inhibitor ± nimotuzumab/capecitabine	5
	None	–	78

### 2.3 Observation indicators

1. Short-term efficacy: A comprehensive re-examination was conducted 3 weeks after the completion of induction therapy and 3 months after the completion of CCRT to assess the short-term efficacy. The longest diameter of the tumor was measured through imaging examinations, and the response was evaluated according to the internationally accepted Response Evaluation Criteria in Solid Tumors (RECIST) version 1.1, based on the change in the longest diameter of the tumor post-treatment compared to baseline. The efficacy assessment included Complete Response (CR), Partial Response (PR), Stable Disease (SD), and Progressive Disease (PD). The Objective Response Rate (ORR) was defined as the proportion of CR + PR.
2. Short-term AEs: AEs occurring during the treatment period were recorded, and the Common Terminology Criteria for Adverse Events (CTCAE) version 5.0 was used as the grading standard for AEs.
3. Long-term efficacy: Follow-up was conducted to determine the patients’ local recurrence, distant metastasis, and survival status. The primary endpoint was Progression-Free Survival (PFS), and the secondary endpoints were Distant Metastasis-Free Survival (DMFS), Overall Survival (OS), and Locoregional Relapse-Free Survival (LRFS).

### 2.4 Follow-up

Patients were scheduled for follow-up examinations every 3 months within the first 2 years post-treatment, every 6 months from 2 to 5 years, and annually thereafter. The follow-up period concluded on October 31, 2024, with a duration ranging from 1 to 123 months and a median follow-up time of 22 months.

### 2.5 Statistical analysis

Statistical analysis was performed using SPSS 22.0 software. Categorical data were expressed as numbers (percentages) [n (%)], and comparisons between groups were conducted using the Chi-square test (χ² test) or Fisher’s exact test. Survival curves were plotted using the Kaplan-Meier method, and differences in survival between groups were compared using the Log-rank test. This study employed a 1:1 propensity score-matched design to enhance comparability between the ICI-containing and standard therapy groups. Matching was based on key baseline characteristics including age, gender, TNM stage (AJCC 8th edition), and clinical stage. Given the limited sample size and the low number of events for key endpoints, the statistical power for reliable multivariable Cox regression modeling was deemed insufficient. Therefore, the presented survival comparisons should be interpreted as exploratory and descriptive of associations, rather than as evidence of independent treatment effects. A P-value of <0.05 was considered statistically significant. All statistical analyses were two-tailed to ensure the scientific validity and reliability of the results.

## 3 Result

### 3.1 Clinical features and survival outcomes

Among 102 stage III–IVB CAYA NPC patients, the median age was 17 years (range: 6–24). Radiation dose data were unavailable for two patients, and baseline EBV-DNA levels were missing in 23 cases. Additional clinical characteristics are comprehensively summarized in **Table 2**.

**Table 2:** Clinical characteristics of 102 patients with stage III-IVB CAYA NPC.

Characteristic	Number of cases (n=102)
Gender	
Male	72
Female	30
Age	
6∼12y	13
13∼18y	47
19y∼24y	42
Induction Regimen	
Chemotherapy	87
TPF	20
TP	40
GP	25
PF	2
ICIs combined with chemotherapy	15
Radiotherapy Regimen	
Chemoradiotherapy	61
Radiotherapy + anti-EGFR therapy	24
Radiotherapy alone	15
Consolidation Therapy	
Yes	24
Chemotherapy	19
ICIs-based therapy	5
No	78
Initial EBV-DNA Level	
Positive (≥500 copies/ml)	30
Negative (<500 copies/ml)	49

At a median follow-up of 22 months, the 2-year OS rate was 97.4% (95% CI: 92.3–100%). However, due to an insufficient number of events (n=2) and the relatively short follow-up, the median OS and 3-year OS rate were not reached. The 2-year PFS rate was 77.3% (95% CI: 68.3–86.3%), the 2-year LRFS rate was 93.4% (95% CI: 87.7–99.1%), and the 2-year DMFS rate was 83.7% (95% CI: 76.1–91.3%).

### 3.2 Efficacy and safety analysis of induction therapy

#### 3.2.1 Efficacy and safety analysis of different induction chemotherapy regimens

**Figure 1:**
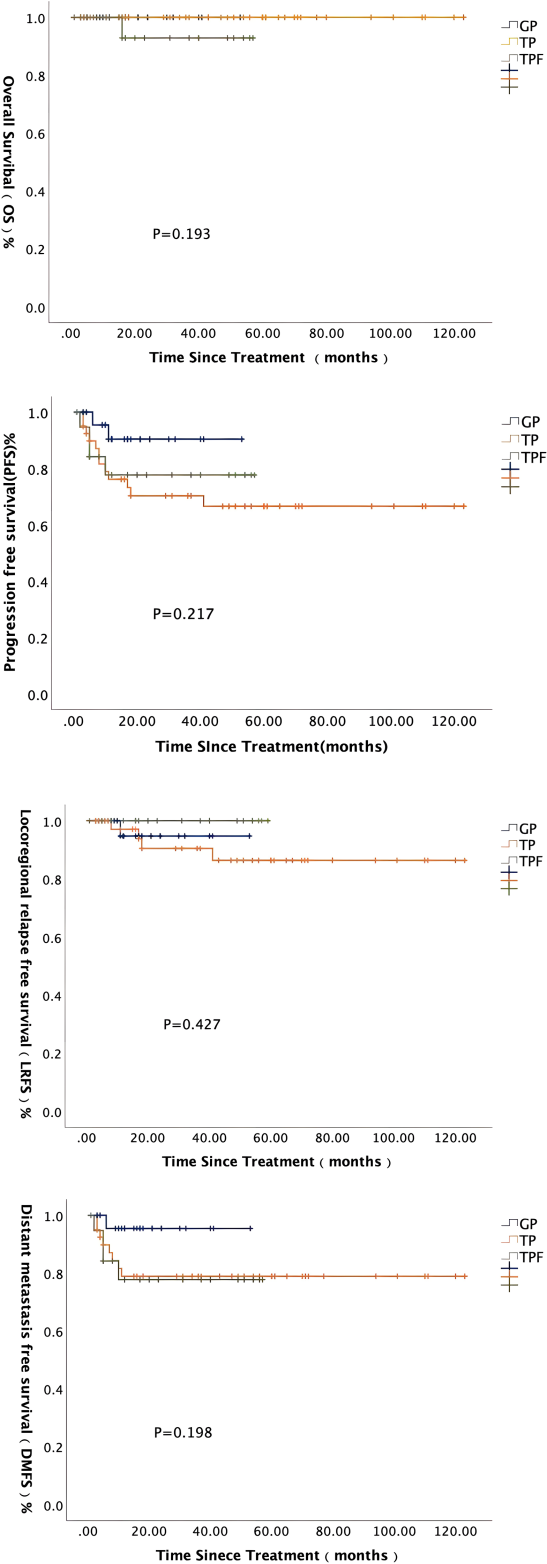
Survival curves of different induction chemotherapy regimens.

Among the 102 patients who received induction therapy, 87 underwent induction chemotherapy, including 20 cases with the TPF regimen, 25 cases with the GP regimen, 40 cases with the TP regimen, and 2 cases with the PF regimen. Three patients did not undergo efficacy evaluation due to personal reasons. The objective response rates (ORR) after induction chemotherapy in the TPF, GP, and TP groups were 89.5%, 68.0%, and 31.6%, respectively (**Table 3**). There were significant statistical differences in tumor response between the GP and TP groups, as well as between the TPF and TP groups. These results indicate that while both GP and TPF regimens were significantly more effective than TP in achieving initial tumor shrinkage, this superior short-term response did not translate into a detectable difference in long-term survival outcomes within the current follow-up period (**Figure 1**).

**Table 3:** AEs in GP and TPF Groups during induction chemotherapy.

Adverse Event	GP (n=25, %)	TPF (n=20, %)	X^2^	P
Leukopenia			8.83	0.009
Grade 1-2	4 (16.0)	3 (15.0)		
Grade 3-4	0	6 (30.0)		
Neutropenia			0.46	0.910
Grade 1-2	3 (12.0)	3 (15.0)		
Grade 3-4	5 (20.0)	5 (25.0)		
Thrombocytopenia			1.42	0.768
Grade 1-2	1 (4.0)	2 (10.0)		
Grade 3-4	1 (4.0)	0		
Gastrointestinal disorders			5.74	0.052
Grade 1-2	15 (60.0)	5 (25.0)		
Grade 3-4	2 (8.0)	2 (10.0)		
Hepatic dysfunction			1.56	0.603
Grade 1-2	6 (24.0)	6 (30.0)		
Grade 3-4	0	1 (5.0)		
Fatigue			0.64	0.423
Grade 1-2	1 (4.0)	2 (10.0)		
Grade 3-4	0	0		
Rash			0.03	0.872
Grade 1-2	1 (4.0)	1 (5.0)		
Grade 3-4	0	0		
Hair loss			0.026	0.872
Grade 1-2	1 (4.0)	1 (5.0)		
Grade 3-4	0	0		

The Common Terminology Criteria for adverse events (CTCAE, version 5.0) was used as the grading standard for AEs, categorizing chemotherapy-related AEs into Grade I, II, III, and IV. In terms of safety, the incidence of leukopenia in the GP group was significantly lower than that in the TPF group (16.0% vs. 45.0%, P = 0.009). The incidence of hepatic dysfunction in the GP group was significantly higher than that in the TP group (24.0% vs. 5.0%, P = 0.011). The incidences of leukopenia, thrombocytopenia, and hepatic dysfunction in the TP group were significantly lower than those in the TPF group (20.0% vs. 45.0%, P = 0.009; 0.0% vs. 10.0%, P = 0.042; 5.0% vs. 35.0%, P = 0.002, respectively). However, the incidence of gastrointestinal disorders in the TP group was significantly higher than that in the TPF group (70.0% vs. 35.0%, P = 0.010) (**Tables 3**–**5**).

**Table 4:** AEs in GP and TP groups during induction chemotherapy.

Adverse Event	GP (n=25,%)	TP (n=40,%)	X^2^	P
Leukopenia			0.67	0.999
Grade 1-2	4 (16.0)	7 (17.5)		
Grade 3-4	0	1 (2.5)		
Neutropenia			1.60	0.514
Grade 1-2	3 (12.0)	10 (25.0)		
Grade 3-4	5 (20.0)	7 (17.5)		
Thrombocytopenia			3.11	0.144
Grade 1-2	1 (4.0)	0		
Grade 3-4	1 (4.0)	0		
Gastrointestinal disorders			0.504	0.920
Grade 1-2	15 (60.0)	26 (65.0)		
Grade 3-4	2 (8.0)	2 (5.0)		
Hepatic dysfunction			7.36	0.011
Grade 1-2	6 (24.0)	1 (2.5)		
Grade 3-4	0	1 (2.5)		
Fatigue			0.33	0.568
Grade 1-2	1 (4.0)	3 (7.5)		
Grade 3-4	0	0		
Rash			0.12	0.733
Grade 1-2	1 (4.0)	1 (2.5.)		
Grade 3-4	0	0		
Hair loss			0.33	0.568
Grade 1-2	1 (4.0)	3 (7.5)		
Grade 3-4	0	0		
Fever			1.29	0.256
Grade 1-2	0	2 (5.0)		
Grade 3-4	0	0		

**Table 5:** AEs in TP and TPF groups during induction chemotherapy.

Adverse Event	TP (n=40, %)	TPF (n=20, %)	X^2^	P
Leukopenia			8.95	0.009
Grade 1-2	7 (17.5.)	3 (15.0)		
Grade 3-4	1 (2.5)	6 (30.0)		
Neutropenia			1.02	0.648
Grade 1-2	10 (25.0)	3 (15.0)		
Grade 3-4	7 (17.5)	5 (25.0)		
Thrombocytopenia			4.14	0.042
Grade 1-2	0	2 (10.0)		
Grade 3-4	0	0		
Gastrointestinal disorders			8.73	0.010
Grade 1-2	26 (65.0)	5 (25.0)		
Grade 3-4	2 (5.0)	2 (10.0)		
Hepatic dysfunction			9.74	0.002
Grade 1-2	1 (2.5)	6 (30.0)		
Grade 3-4	1 (2.5)	1 (5.0)		
Fatigue			0.11	0.741
Grade 1-2	3 (7.5)	2 (10.0)		
Grade 3-4	0	0		
Rash			0.26	0.611
Grade 1-2	1 (2.5)	1 (5.0)		
Grade 3-4	0	0		
Hair loss			0.13	0.714
Grade 1-2	3 (7.5)	1 (5.0)		
Grade 3-4	0	0		
Fever			1.03	0.309
Grade 1-2	2 (5.0)	0		
Grade 3-4	0	0		

#### 3.2.2 Efficacy and safety analysis of induction therapies containing ICIs

This study employed a 1:1 propensity score-matched design to control for the potential influence of confounding factors. The matching variables included baseline characteristics such as patient age, gender, TNM stage (based on the AJCC 8th edition staging criteria), clinical stage, and histological grade. After rigorous matching, a total of 30 patients were enrolled, with 15 patients in the ICIs group and 15 in the standard therapy group. Statistical analysis revealed no significant differences in baseline characteristics between the two groups (P > 0.05), indicating good comparability. Detailed data are presented in **Table 6**.

**Table 6:** Clinical characteristics of 30 matched patients with stage III-IVB NPC receiving induction therapy with and without ICIs.

Characteristic	ICIs group (n=15)	Standard therapy group (n=15)	X^2^	P
Gender			0.60	0.439
Male	9	4		
Female	6	11		
Age			0.44	0.801
6∼12y	2	2		
13∼18y	4	5		
19y∼24y	9	9		
ECOG			2.41	0.299
0	6	4		
1	4	10		
2	1	1		
T Stage			2.48	0.48
1	1	1		
2	1	1		
3	8	4		
4	5	9		
N Stage			0.14	0.931
1	1	1		
2	7	6		
3	7	8		
M Stage			0.11	0.742
0	11	11		
1	4	3		
Clinical stage			0.00	1.000
III-IVA	11	11		
IVB	4	4		
Histological Grade			1.03	0.309
Differentiated	0	1		
Undifferentiated	15	14		
Initial EBV-DNA Level			0.05	0.831
Positive (≥500 copies/ml)	6	4		
Negative (<500 copies/ml)	9	5		

**Figure 2:**
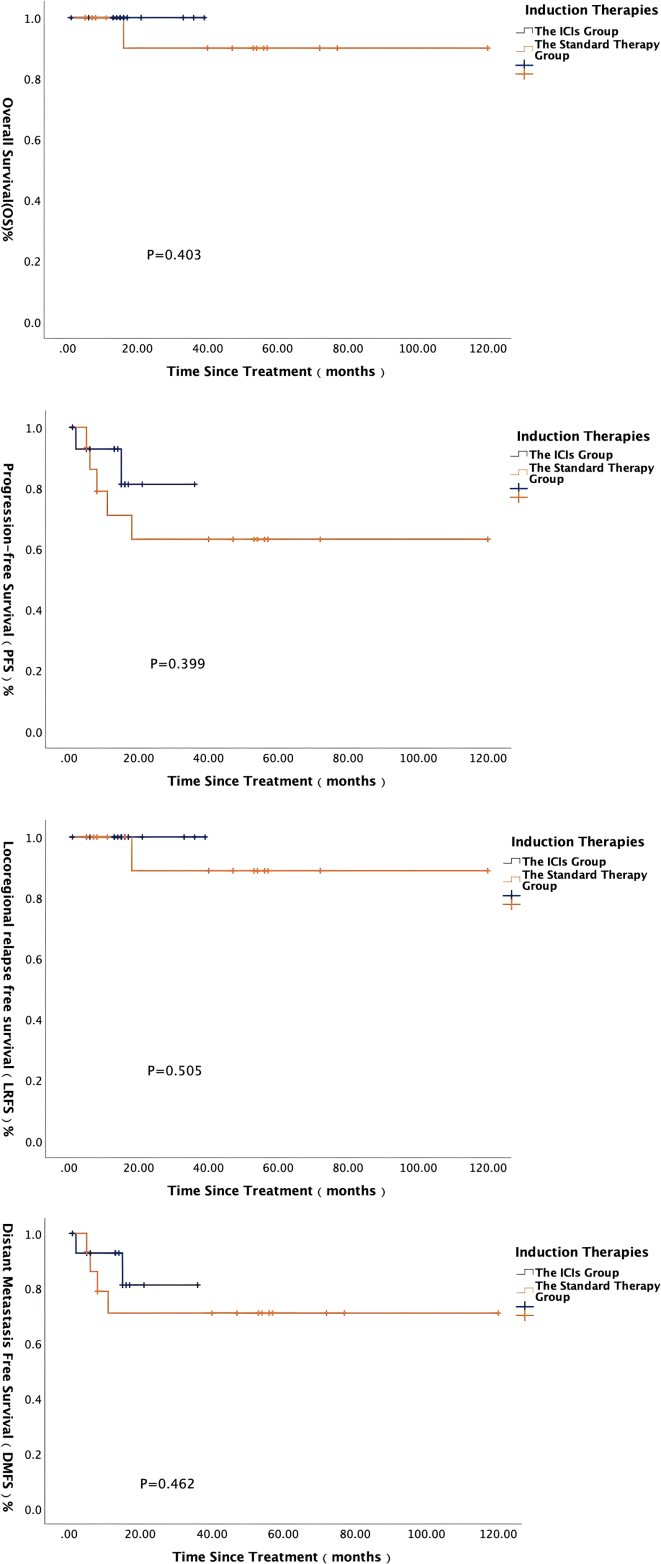
Survival curves of 30 matched patients with stage III-IVB NPC receiving induction therapy with and without ICIs.

In terms of the induction treatment regimen, the ICIs group received TP/GP regimens combined with PD-1 inhibitor (± nimotuzumab). Efficacy evaluation showed that the ORR in the ICIs group was 93.3%, significantly higher than the 53.3% in the standard therapy group (P = 0.013). For long-term prognosis, Kaplan-Meier survival analysis was performed for OS, PFS, LRFS, and DMFS in both groups (**Figure 2**). This finding demonstrates that the addition of ICIs to induction chemotherapy markedly improved the rate of objective tumor response. However, within the limits of the present follow-up and sample size, this enhanced response rate was not associated with a statistically significant improvement in survival endpoints (P > 0.05), highlighting a need for longer-term observation.

We documented the incidence of immune-related AEs in 15 patients who received induction therapy containing ICIs. The results showed that hepatic dysfunction was the most common immune-related adverse reaction, with an incidence rate of 33.3% (5/15). Among these cases, Grade I-II hepatic dysfunction accounted for 26.7% (4/15), and Grade III accounted for 6.7% (1/15). One patient discontinued ICIs-therapy prematurely due to severe hepatic dysfunction (see **Table 7** for details).

**Table 7:** Incidence of IRAEs in 15 matched patients receiving ICIs.

Immune related adverse event	Grade I-II (%)	Grade III-IV (%)
Hepatic dysfunction	4 (26.7)	1 (6.7)
Hypothyroidism	3 (20.0)	0
Rash	2 (13.3)	0
Myocardial injury	1 (6.7)	0

#### 3.2.3 The impact of post-induction efficacy on long-term survival

**Figure 3:**
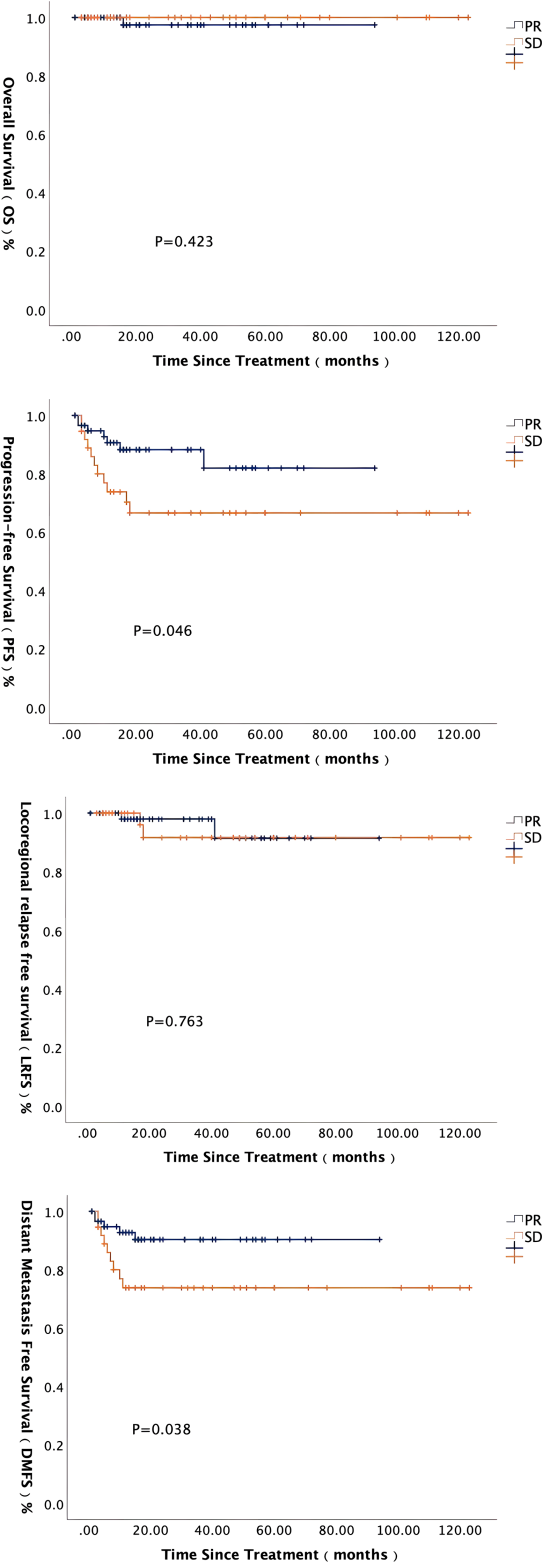
Long-term survival analysis based on different post-induction efficacy.

Among the 102 enrolled patients, 4 were unable to complete efficacy evaluation due to personal reasons, and ultimately 98 patients were included in the efficacy analysis. According to the Response Evaluation Criteria in Solid Tumors (RECIST 1.1), patients were divided into the PR group (60/98, 61.2%) and the SD group (38/98, 38.7%) based on their response after induction therapy. Long-term survival analysis showed that both PFS and DMFS in the PR group were significantly better than those in the SD group. The 5-year PFS rate in the PR group was 82.0%, significantly higher than the 66.7% in the SD group (P = 0.046). The 5-year DMFS rate in the PR group was 90.3%, also significantly higher than the 73.8% in the SD group (P = 0.038). For details, see **Figure 3**.

### 3.3 Efficacy analysis of radiotherapy combined with chemotherapy/anti-EGFR therapy

**Figure 4:**
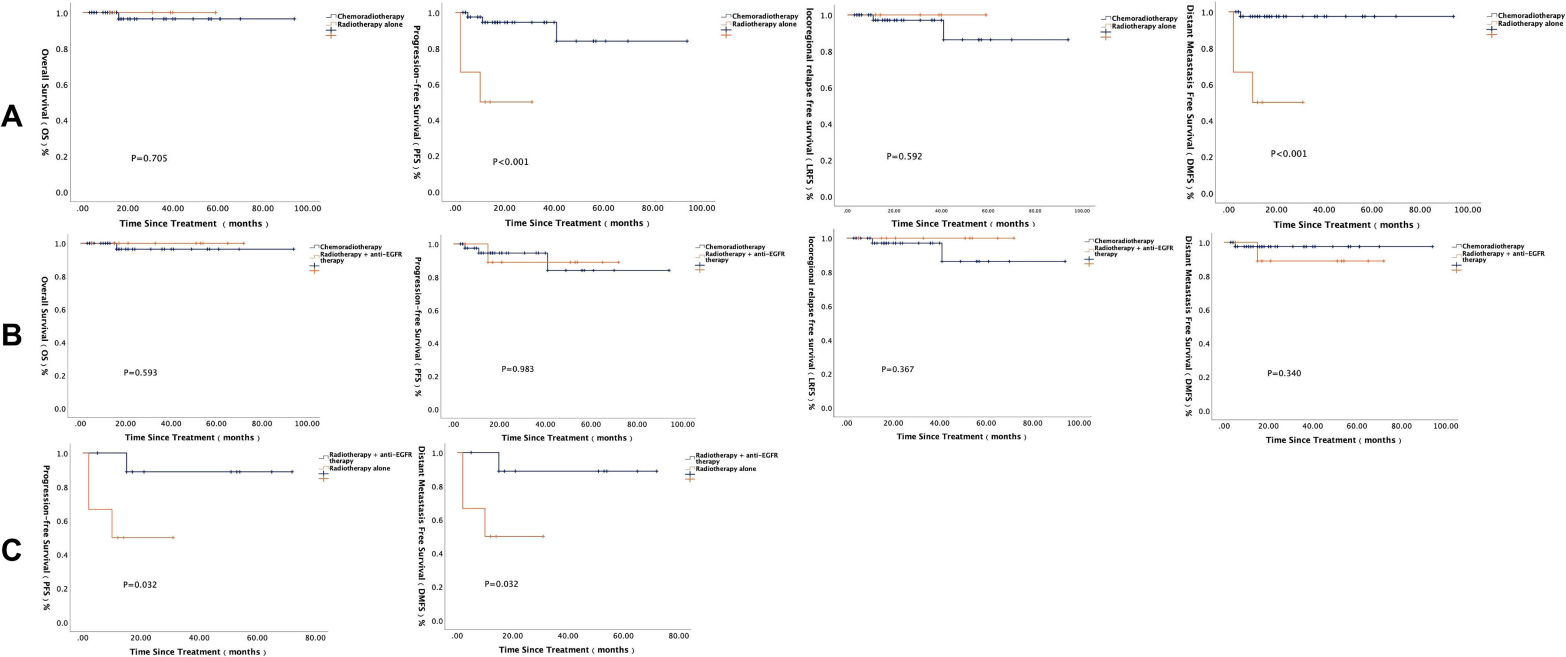
Survival analysis of different radiotherapy regimens in patients with PR after induction therapy.

**Figure 5:**
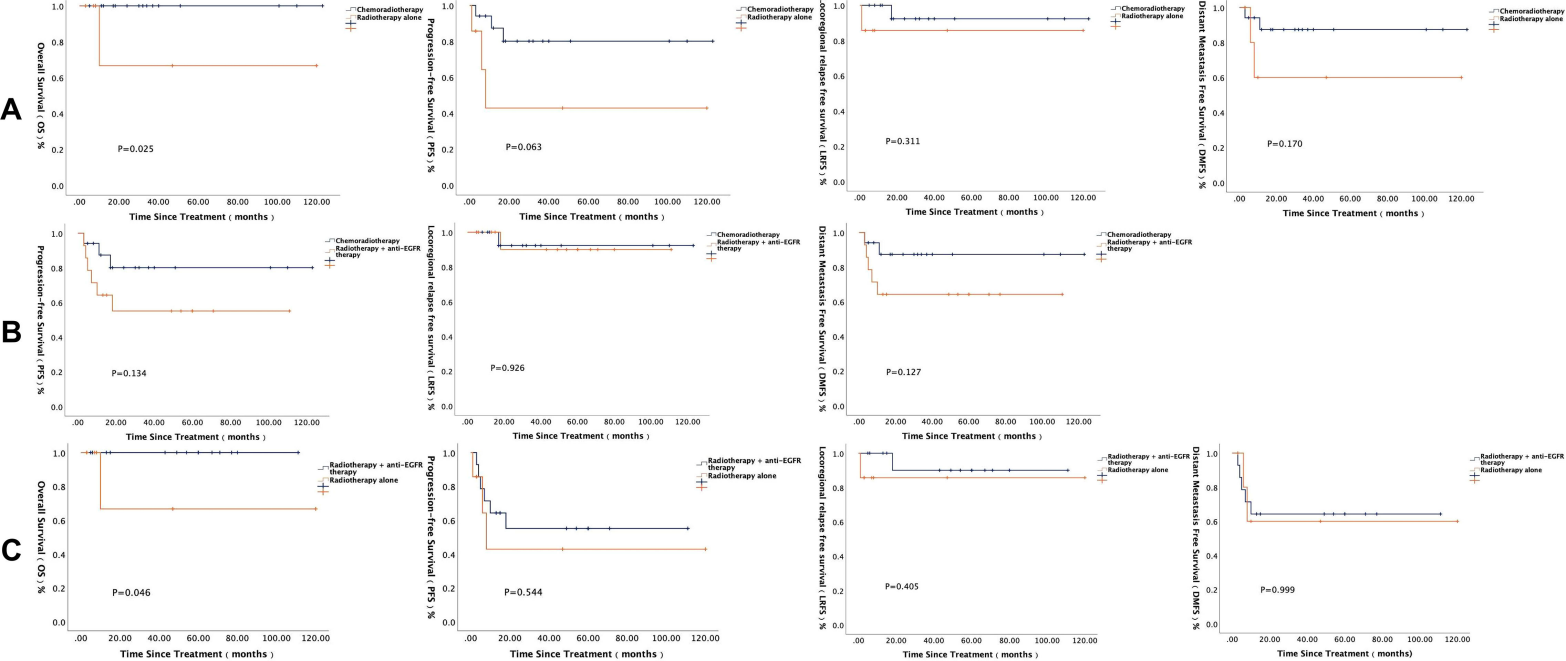
Survival analysis of different radiotherapy regimens in patients with SD after induction therapy.

During radiotherapy, 61 patients received combined chemotherapy, 24 patients received combined anti-EGFR therapy, and 15 patients received radiotherapy alone. The differences in long-term survival among the groups are shown in **Figures 4** and **5**. In the subgroup of patients who achieved PR after induction therapy, survival analysis results showed that the 1-year PFS and DMFS rates in the combined anti-EGFR therapy group were both 88.9%, significantly better than those in the radiotherapy-alone group. Additionally, the 1-year PFS and DMFS rates in the combined chemotherapy group were 94.5% and 97.4%, respectively, significantly higher than those in the radiotherapy-alone group. In the subgroup of patients who achieved SD after induction therapy, the 5-year OS rate in the combined anti-EGFR therapy group was 100%, significantly higher than the 66.7% in the radiotherapy-alone group. The 5-year OS rate in the combined chemotherapy group was also 100%, significantly higher than the 66.7% in the radiotherapy-alone group.

### 3.4 Efficacy of consolidation therapy

**Figure 6:**
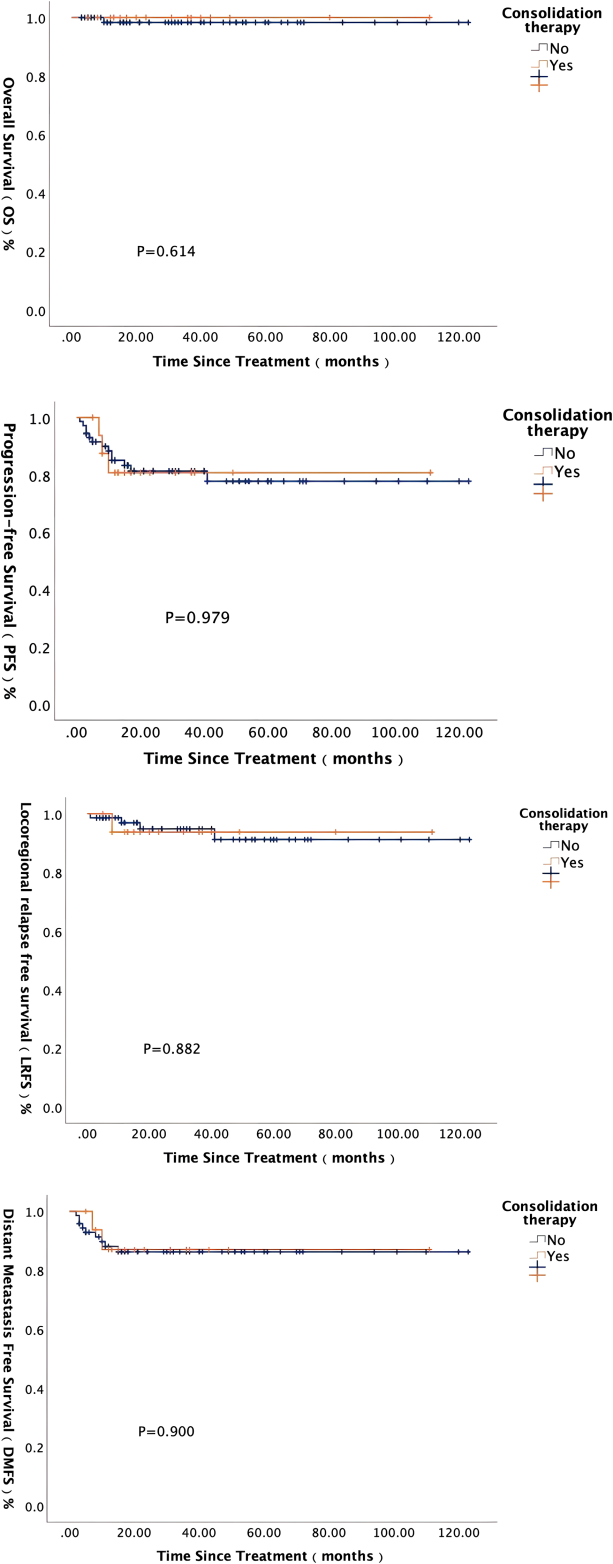
Long-term survival analysis of locally advanced NPC patients receiving consolidation therapy.

Among the 102 patients in this study, follow-up data from 98 patients included information on post-radiotherapy treatment, of which 24 patients received consolidation therapy. Five patients received consolidation therapy with PD-1 inhibitor ± nimotuzumab/capecitabine, while the remaining 19 patients underwent chemotherapy regimens, including GP/TPF/TP/PF/capecitabine. Notably, among advanced-stage (IVB) patients, 7 patients (7/9, 77.8%) received consolidation therapy. Further analysis showed that clinical stage was a significant factor influencing the choice of consolidation therapy (P = 0.001, 95% CI 0.000-0.001). Based on these results, we conducted long-term survival analysis on 89 patients with locally advanced (III-IVA) NPC. All patients had negative EBV-DNA levels before consolidation therapy, and only 1 patient experienced disease progression prior to consolidation therapy. There were no significant differences in baseline clinical characteristics among the groups (**Table 8**). Survival analysis results (**Figure 6**) showed that in locally advanced NPC patients, consolidation therapy did not significantly impact long-term survival outcomes at this early follow-up (P > 0.05). Therefore, within the context of our study cohort and follow-up duration, the routine use of consolidation therapy did not confer a demonstrable survival benefit for patients with locally advanced disease.

**Table 8:** Clinical characteristics of locally advanced (III-IVA) CAYA NPC patients.

Characteristic	Non-consolidation therapy group (n=72)	Consolidation therapy group (n=17)	X^2^	P
Gender			1.60	0.206
Male	48	14		
Female	24	3		
Age			2.58	0.289
6∼12y	24	9		
13∼18y	35	7		
19y∼24y	13	1		
ECOG			2.76	0.507
0	29	9		
1	38	5		
2	3	0		
T Stage			4.50	0.117
1	1	2		
2	9	1		
3	29	8		
4	33	6		
N Stage			2.49	0.472
0	2	0		
1	4	2		
2	39	11		
3	28	4		
Histological Grade			2.57	0.352
Differentiated	9	0		
Undifferentiated	60	17		
Not clear	1	0		
Evaluation (3 months after radiotherapy)			0.44	0.999
Local control	64	15		
Distant metastasis	2	0		
Local recurrence	4	1		
Evaluation after radiotherapy			3.60	0.237
PR	43	9		
CR	15	0		
SD	4	1		
PD	2	0		
Initial EBV-DNA Level			0.22	0.638
Positive (≥500 copies/ml)	3	0		
Negative (<500 copies/ml)	47	8		

## 4 Discussion

As a retrospective analysis, this study is subject to inherent limitations including selection bias, treatment heterogeneity, and unmeasured confounding factors. Due to the low incidence of CAYA NPC, conducting large-scale prospective clinical studies remains challenging, and treatment strategies are typically guided by adult NPC therapeutic guidelines. For patients with stage I-II (N0) NPC, radiotherapy alone can achieve a favorable survival rate, with a 10-year overall survival (OS) rate reaching 98% (14). In contrast, advanced-stage CAYA NPC patients receiving radiotherapy alone demonstrate poorer prognoses, showing a 4-year DFS rate of only 40% (15). The efficacy and safety of combining radiotherapy with chemotherapy have been well validated in adult NPC patients (16). Furthermore, multiple retrospective studies indicate that combined-modality therapy significantly improves survival rates in CAYA NPC patients. Research by Cheuck et al. demonstrated a 15-year OS of 81% for patients treated with radiotherapy combined with cisplatin, compared to 54% in the radiotherapy-alone group (17). Prospective studies focusing on stage II (N1), III, and IVA CAYA NPC patients further confirm that combined therapy markedly enhances survival outcomes, establishing it as the standard treatment for this population (5, 18–20). For stage IVB NPC patients, multimodal therapeutic strategies—including induction chemotherapy, radiotherapy, local metastasis-directed therapy, and consolidation therapy—have been widely adopted (5, 21). This study demonstrated significant survival benefits of combined therapy in III-IVB stage CAYA NPC patients. The entire cohort achieved an excellent 2-year overall survival rate of 97.4%, with particularly outstanding locoregional control (LRFS: 93.4%). Although the progression-free survival (PFS: 77.3%) and distant metastasis-free survival rate (DMFS: 83.7%) were relatively lower, the overall survival outcomes remained significantly superior to historical controls. However, given the limited median follow-up of 22 months and few events (n=2), extended follow-up is warranted to evaluate long-term survival outcomes, and the findings should be interpreted cautiously.

Previous studies have demonstrated significant differences in efficacy and AEs among induction chemotherapy regimens for adult NPC patients. In the treatment of locally advanced NPC, the GP regimen has shown superior efficacy compared to the PF regimen (22). Two phase III trials published in 2016 and 2019 revealed that TPF or GP induction chemotherapy prior to concurrent chemoradiotherapy significantly improved survival outcomes in adults with locally advanced NPC (7, 23). However, in pediatric and adolescent populations, multiple studies have reported no statistically significant differences in induction efficacy or long-term survival across regimens. The NCT00565448 trial compared PF and TPF induction chemotherapy in NPC patients under 21 years of age, with survival analysis showing no significant intergroup differences (3-year OS: 78.0% vs. 85.7%, respectively) (24). Research by Ou et al. further indicated comparable long-term survival and disease control rates among adolescent NPC patients receiving various induction regimens (GP, TP, TPF, PF) (25).

In this study, no significant differences in long-term outcomes were observed among CAYA NPC patients treated with different induction regimens. However, patients receiving GP or TPF induction achieved significantly higher partial response rates post-induction compared to those treated with TP (GP vs. TP: 68% vs. 31.6%, P = 0.005; TPF vs. TP: 89.5% vs. 31.6%, P<0.001). No statistically significant difference in partial response rates was noted between GP and TPF regimens (GP vs. TPF: 68% vs. 89.5%, P = 0.092). Regarding safety, the incidence of leukopenia was significantly lower in the GP group than in the TPF group. Therefore, the GP regimen may be recommended as the preferred induction chemotherapy for stage III-IVB CAYA NPC patients.

In NPC endemic regions, including China, the development and progression of NPC are closely associated with EBV infection. EBV-associated NPC exhibits a high PD-L1 positivity rate (approximately 95%) and dense lymphocytic infiltration within the tumor microenvironment. Mechanistically, EBV-encoded latent membrane protein 1 (LMP1) and interferon-gamma (IFN-γ) signaling pathways upregulate PD-L1 expression in NPC, providing a robust theoretical foundation for immunotherapy in this malignancy (5). Furthermore, multiple phase III clinical trials have demonstrated that combining PD-1 inhibitors with GP chemotherapy as first-line therapy significantly improves progression-free survival (PFS) in recurrent/metastatic NPC patients, reinforcing the clinical efficacy of immunotherapy in NPC management (26–28). Whole-exome sequencing of blood specimens from CAYA NPC patients revealed significantly higher PD-L1 expression levels compared to adult NPC patients (percentage of patients with PD-L1 expression >50%: 92.0% vs. 32.1%), suggesting a potential therapeutic benefit from ICIs in this population (29).

In this study, patients receiving combined ICIs induction achieved significantly higher response rates compared to chemotherapy-alone induction (93.3% vs. 53.3%, P < 0.05). However, this enhancement in tumor response did not translate into a statistically significant improvement in progression-free or overall survival in our matched analysis at this follow-up. This underscores the complex relationship between initial tumor response and ultimate survival outcomes, and highlights the need for longer follow-up and larger studies to determine if the higher response rates conferred by ICIs will lead to enduring survival advantages in the CAYA population. Regarding safety, only 1 of 15 patients (6.7%) in the ICIs group experienced grade 3–4 immune-related adverse events (irAEs), indicating favorable tolerability in CAYA NPC populations.

Additionally, this study evaluated the prognostic impact of induction therapy responses. Patients achieving PR post-induction showed significantly superior 5-year PFS and DMFS compared to those with SD status (82.0% vs. 66.7%; 90.3% vs. 73.8%, respectively). Consistent with prior studies (15, 30, 31), reducing radiotherapy doses for induction responders may mitigate late toxicities without compromising locoregional control or long-term survival outcomes.

Based on the aforementioned research findings, we hypothesize that the use of PD-1 inhibitor combined with the GP regimen as induction therapy in CAYA NPC patients may enhance disease response rates, potentially reducing late-onset toxicities, and consequently improving PFS and DMFS. However, it should be emphasized that this study is limited by its retrospective design and the preliminary nature of the survival data. Therefore, this therapeutic strategy requires further validation through large-scale, multicenter, randomized controlled prospective studies.

Over the past three decades, multiple phase III clinical trials have consistently validated the efficacy of CCRT in NPC management. A meta-analysis published in The Lancet Oncology further solidified CCRT as the standard treatment for NPC. This analysis, encompassing 19 studies and 4,806 patients, demonstrated that CCRT significantly improved OS (HR = 0.79, 95% CI 0.73–0.86, P < 0.0001), with a 5-year absolute OS benefit of 6.3% (95% CI 3.5–9.1). Additionally, 5-year PFS increased from 59.8% to 69.9%, representing an absolute gain of 10.1% (32). However, a multicenter phase III trial revealed that for locoregionally advanced NPC patients, radiotherapy alone following induction chemotherapy achieved non-inferior 3-year PFS compared to CCRT (76.2% vs. 76.8%, HR = 0.92, 95% CI 0.65- 1.32, P =0.66). The radiotherapy-alone group exhibited lower incidences of grade 3–4 acute toxicities than the CCRT group, though late toxicities were comparable between arms (33).

In our study, among patients achieving PR post-induction, significant differences in OS and distant DMFS were observed between the CCRT and radiotherapy-alone groups, with 1-year OS and DMFS rates both at 88.9%. Similarly, the CCRT group showed superior 1-year PFS (94.5% vs. 50%) and DMFS (97.4% vs. 50%) compared to radiotherapy alone. For patients with SD post-induction, CCRT significantly improved 5-year OS (100% vs. 66.7%). These findings suggest that CCRT yields superior long-term outcomes compared to radiotherapy alone, regardless of whether patients achieve PR or SD before definitive radiotherapy, providing critical evidence for optimizing clinical strategies.

For advanced metastatic NPC patients without progression after first-line GP chemotherapy combined with PD-1 inhibitors, PD-1 monotherapy maintenance is recommended until intolerable toxicity, disease progression, or completion of 2 years (27, 34). Patients receiving chemotherapy alone may consider maintenance therapy with low-toxicity oral fluoropyrimidines (e.g., capecitabine, S-1). However, the role of consolidation therapy in locoregionally advanced NPC remains controversial (35). A major concern is the high incidence of AEs; approximately 40% of patients discontinue consolidation chemotherapy due to severe mucositis or weight loss. Previous studies found no survival benefit with high-dose consolidation chemotherapy (36, 37), while low-dose metronomic chemotherapy improved 3-year OS (38). Prospective trials GPOH-NPC-91 and GPOH-NPC-2003 demonstrated that combining PF induction, CCRT, and interferon-β maintenance achieved OS and PFS rates exceeding 90% in CAYA patients with locoregionally advanced NPC (19, 20).

In this study, survival outcomes did not differ significantly between locoregionally advanced NPC patients receiving or not receiving consolidation therapy. However, the small consolidation cohort (n = 24, including 18 locoregionally advanced cases) may limit the generalizability of these findings. Notably, among the 18 locoregionally advanced patients receiving consolidation, nine achieved PR or better post-CCRT, and all exhibited undetectable EBV-DNA, suggesting potential for omitting consolidation in responders. Due to limited sample size, inherent retrospective biases, and the short follow-up, definitive conclusions require validation through large-scale, multicenter studies aimed at identifying patient subgroups most likely to benefit from consolidation therapy, thereby optimizing therapeutic strategies and improving quality of life in CAYA NPC patients.

The potential prognostic role of plasma EBV-DNA, a well-established biomarker in adult NPC, could not be fully elucidated in our cohort due to missing data for a subset of patients. Future prospective studies in CAYA NPC should prioritize the standardized collection and analysis of EBV-DNA to clarify its utility in this age group.

## 5 Future directions and conclusions

This retrospective study demonstrates that GP and TPF induction chemotherapy regimens, combined with PD-1 inhibitors, improve short-term tumor response rates in locally advanced CAYA NPC patients, with acceptable toxicity profiles. Although immunotherapy enhances objective response rates, its long-term survival benefits require further investigation. The role of consolidation therapy remains uncertain in this cohort, highlighting the need for personalized approaches to minimize unnecessary treatment burdens.

The retrospective nature of this analysis precludes definitive causal inferences. The observed associations, particularly regarding the role of ICIs and consolidation therapy, must be validated in rigorously designed prospective studies. Future studies should focus on large-scale randomized trials to establish standardized induction and consolidation protocols, optimize ICIs-based therapeutic strategies, and identify biomarkers for patient selection. Exploring de-escalation strategies for radiotherapy and chemotherapy in responders, alongside evaluations of long-term toxicities and quality of life, are critical to advancing precision oncology. Collaborative efforts are essential to translate these insights into evidence-based guidelines, ultimately improving outcomes for this vulnerable cohort.

## Data Availability

The raw data supporting the conclusions of this article will be made available by the authors, without undue reservation.
