# Correction: New insights into the treatment of nasopharyngeal carcinoma in children, adolescents, and young adults: a retrospective study

**DOI:** 10.3389/fimmu.2026.1821175

**Published:** 2026-03-10

**Authors:** Mengwei Ren, Jing Tian, Mengyuan Han, Shiran Sun, Kai Wang, Runye Wu, Meng Yuan, Junlin Yi, Fei Ma, Sidan Li

**Affiliations:** 1 Department of Medical Oncology, National Cancer Center/National Clinical Research Center for Cancer/Cancer Hospital, Chinese Academy of Medical Sciences and Peking Union Medical College, Beijing, China; 2 Hematology Center, Beijing Key Laboratory of Pediatric Hematology Oncology; National Key Discipline of Pediatrics (Capital Medical University); Key Laboratory of Major Diseases in Children, Ministry of Education; Beijing Children’s Hospital, Capital Medical University, National Center for Children’s Health, Beijing, China; 3 Department of Pediatrics, Beijing Shijitan Hospital, Capital Medical University, Beijing, China; 4 Department of Radiation Oncology, National Cancer Center/National Clinical Research Center for Cancer/Cancer Hospital, Chinese Academy of Medical Sciences and Peking Union Medical College, Beijing, China

**Keywords:** adolescent and young adult, children, immune checkpoint inhibitors, induction chemotherapy, nasopharyngeal carcinoma, retrospective study

In the published article, there was a mistake in the **Funding** statement.

The **Funding** statement was displayed as “The author(s) declared that financial support was received for this work and/or its publication. This research was supported by the following grants: CAMS Innovation Fund of Medical Sciences (2022-I2M-2-001) (supporting methodological innovation and exploratory research); National Key Research and Development Program of China (2024YFA1107400).”

The correct **Funding** statement reads: “The author(s) declared that financial support was received for this work and/or its publication. This research was supported by the following grants: National Key Research and Development Program of China (2024YFA1107400); The National Natural Science Foundation of China (No. 82370142).”

The original version of this article has been updated.

